# Dispersal patterns of Squamata in the Mediterranean: An evolutionary perspective

**DOI:** 10.1002/ece3.8159

**Published:** 2021-09-24

**Authors:** Daniel Escoriza

**Affiliations:** ^1^ GRECO, Institute of Aquatic Ecology University of Girona Girona Spain

**Keywords:** colonization, island, lizard, phylogeny, snake

## Abstract

Mediterranean islands have a high diversity of squamates, although they are unevenly distributed. This variability in the composition of the reptile assemblages across islands may have been influenced by differences in the colonization abilities of these species. To evaluate the dispersal capacities of squamate species, we modeled their sea routes using cost surface models. We estimated the effects of some life‐history traits and the phylogenetic signal in the characteristics of the modeled dispersal paths. We hypothesized that a significant phylogenetic signal should be present if the dispersal ability is enhanced by traits shared among evolutionarily related species. The results showed that no phylogenetic signal was present in the characteristics of the dispersal paths (i.e., in the distance traveled/bypassed sea depth). Thus, no superior island‐colonizer lineages were detected in Mediterranean Squamata. However, our analyses also revealed that small‐sized lizards were superior to other groups of squamates at dispersing over long distances on the sea.

## Graphical abstract

The dispersal of reptiles throughout the Mediterranean islands lacks a phylogenetic structure. Only small lizards appear to be better dispersers, but this trait evolved independently in several unrelated lineages.

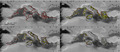

## 1 INTRODUCTION

The Mediterranean islands are populated by rich biotic communities, which comprise a mixture of recently arrived species and ancient insular radiations (Fois et al., 2020). Similar to other regions of the world, the diversity of these island communities has been influenced by geographic isolation and the geophysical characteristics of islands (surface area and topography; Kadmon & Allouche, 2007; MacArthur, 1965). The sea constitutes a powerful barrier to faunal movement, strongly reducing the species that can reach islands (Fattorini, 2002). However, animals can bypass this barrier using intermittent land corridors, or across the sea by swimming or by drifting/floating objects (Spennemann, 2020; Stankiewicz et al., 2006).

The colonization routes followed to reach an island determine the composition of its faunal assemblages. The formation of land corridors allows the almost barrier‐free flow of continental fauna, whereas sweepstake dispersal routes are associated with strong filtering and species poor assemblages (Mazza et al., 2013; Simpson, 1940). The great faunal diversity of the communities on Mediterranean islands may be explained by the fact that species reached the islands using several dispersal routes (Poulakakis et al., 2013). However, unlike other regions of the world (e.g., in oceanic islands, such as the Galapagos or Samoa, in which natural colonization from the mainland probably occurred by rafting dispersal; Caccone et al., 1999; Gill, 1993), it is possible that dispersal among the Mediterranean islands through land corridors played a very important role during the colonization process given that this sea is a closed basin.

The importance of dispersal through land corridors is also supported by phylogeographic studies, which suggested that there is a link between island connectivity and the molecular divergence of subpopulations of species/genera across island systems (Kornilios et al., 2019; Thompson, 1999). Colonization following the formation of land bridges occurred during several phases from the Middle Pliocene (17 Mya) to the later glacial eustatic regressions (20 Kya; Parmakelis et al., 2006). However, some islands such as Mallorca, Crete, and Cyprus are surrounded by deep sea regions, and their isolation has possibly remained uninterrupted since the Messinian event (5.33 Mya; Palombo, 2018).

**FIGURE 1:**
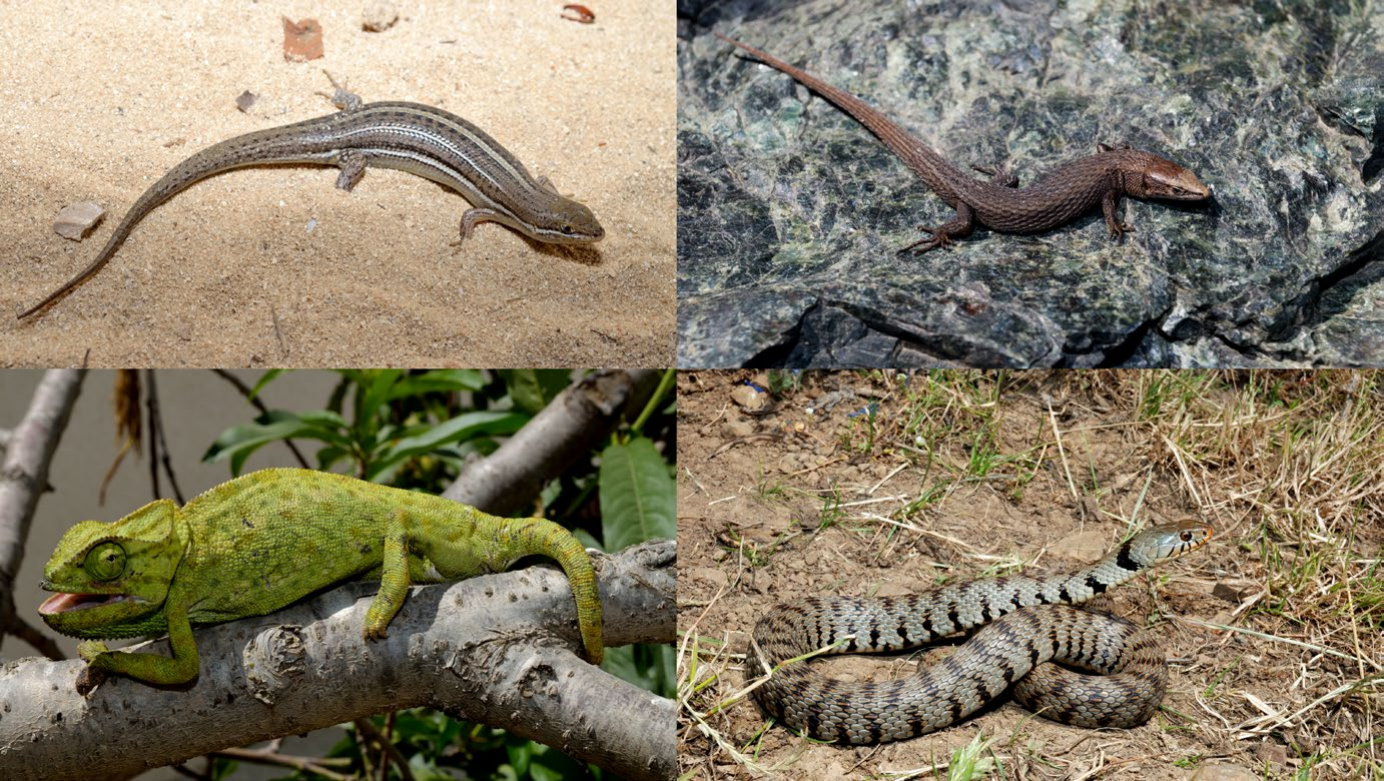
Examples of island reptiles in the Mediterranean. Clockwise: *Algyroides fitzingeri* (Corsica); *Natrix helvetica sicula* (Sicily); *Chamaeleo chamaeleon* (Tunisia); *Heremites vittatus* (Tunisia). Credits: Daniel Escoriza

In this study, we evaluated the dispersal patterns of several species of Squamata (Reptilia) throughout the Mediterranean islands. These islands have relatively diverse reptile assemblages, although this species diversity depends greatly on the sizes of the islands and their geographical locations (Chondropoulos, 1986; Mayol, 1997; Figure 1). Most of the squamate island species only occur on a few islands or a single archipelago, thereby indicating a limited ability to disperse across the sea (Hurston et al., 2009). However, a few species have spread among several archipelagos, such as some geckoes, lacertid lizards, and skinks (Di Nicola & Mezzadri, 2018; Stille et al., 2021), and thus, these species may possess adaptive traits that enhance their capacity as island colonizers. If these traits are only shared among evolutionarily related species, their dispersion patterns should exhibit a phylogenetic signal (hypothesis i). We also tested (hypothesis ii) that squamates with small body sizes and located at lower trophic levels would have been more successful at dispersing across the sea because of their higher capacity to occupy islands with a range of sizes (Holt et al., 1999; Krysko & MacKenzie‐Krysko, 2016; Lomolino, 2005).

## 2 MATERIALS AND METHODS

### 2.1 Study region

**FIGURE 2:**
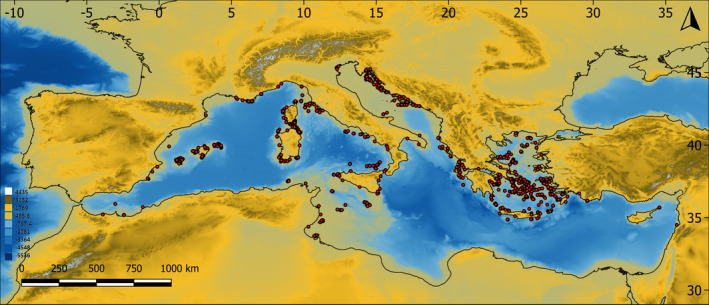
Map of the study region, showing the islands included in the study (red dots)

The study region covered the Mediterranean basin (Figure 2). This basin includes a large number of islands, with broad variability in their isolation and geophysical characteristics (Arnold, 2008; Itescu et al., 2018). In total, 105 species of Squamata occur on these islands and their patterns of occurrence were evaluated based on data obtained from biogeographic atlases and scientific papers (see the references provided in Appendix S1). Squamate species have been separated into endemic and mainland in origin based on recent phylogeographic studies (Kindler et al., 2013; Kornilios et al., 2010, 2019; Kotsakiozi et al., 2018; Senczuk et al., 2019; Spilani et al., 2019; Stöck et al., 2016; Utiger & Schätti, 2004).

### 2.2 Dispersal paths

The dispersal patterns were analyzed by decomposing the paths into two components: length of the route (= distance traveled) and depth of the sea floor along the route (= bypassed sea depth). These two components described the progressive difficulty of reaching an island across the sea (Heaney et al., 2005). The distance traveled was estimated with a minimum‐cost vector network by connecting the vertices of a graph (Sessions, 1992). This procedure generated the shortest path that connected the island network without assuming any dispersal step (i.e., origin–destination). The distance traveled was also estimated by building a cost surface model, which assumed a direction of dispersal (origin–destination) and that the path followed the gradient of minimum resistance (Carroll et al., 2012).

We defined the origins in a different manner for island endemics and mainland species. For endemic species, we considered the origin as the largest central island (e.g., Ibiza for *Podarcis pityusensis* or Crete for *Podarcis cretensis*) or pairs of large central islands (Corsica‐Sardinia or Mallorca‐Menorca) within an archipelago (because endemic species are not restricted to a single island; Speybroeck et al., 2016). This definition is supported by the dispersal patterns described for some endemic species (e.g., *Podarcis lilfordi*, Terrasa et al., 2004). In mainland species, we considered the origin as the continental region nearest to the island where these species are distributed, except for those where the origin has been restricted by phylogeographic studies, such as *Hemidactylus turcicus*, *Tarentola mauritanica,* and *Chalcides ocellatus* (Kornilios et al., 2010; Rato et al., 2010, 2011). In these latter species, only the regions where they are supposed to be native have been considered in the models. The regions of origin were mapped based on Schleich et al. (1996), Geniez (2015), Speybroeck et al. (2016), and IUCN (2021).

The surface resistance was modeled to minimize the traveling cost following the coastline by using three variables: the distance to the coast (greater resistance with distance), terrain elevation (greater resistance with elevation), and depth of the sea floor (greater resistance with sea depth). However, there is still some uncertainty regarding the level of resistance that the sea imposes on the movement of species, and this uncertainty was considered by building three models that assumed various levels of resistance (Beier et al., 2009). Model 1 (M1) assumed that the transmarine route imposed greater resistance than land, although it is still relatively easy to travel across the sea (e.g., in semi‐aquatic snakes, or by drifting on wind‐driven sea currents; Baker, 2015; Renner, 2004). Model 2 (M2) assumed that island colonization occurred mostly during recent eustatic regressions. Species could only travel along the transmarine route until they reached a depth threshold of around −150 m (i.e., minimum sea level during the last glacial cycle; Lambeck & Purcell, 2005). Model 3 (M3) assumed that the transmarine route was very unlikely, so species maximized their movement across land routes, even by significantly increasing the distances traveled.

These models allowed us to generate a network of paths connecting the islands to each other and to the mainland, and we estimated the following variables from these paths: total distance traveled, average distance traveled, maximum distance traveled in a single trip (i.e., connecting two adjacent points), average sea depth along the trip, maximum sea depth along the trip, and percentage of trip above −150 m sea level (Table 1). The mean value of these variables for each model (M1, M2, M3) and species are shown in Appendix S2. The cost surface models and species paths were generated using a digital elevation model of the sea floor (Becker et al., 2009) and the package GRASS‐GIS (GRASS Development Team, 2020).

**TABLE 1:** Description of the variables and descriptive statistics

Variable	Category	Description	Mean	*SE*
Minimum tree	Distance	Minimum‐cost vector network	986.32 km	135.06
Total travel	Distance	Summation of travel lengths	815.59 km	142.83
Average travel	Distance	Total travel/number of travels	53.55 km	9.11
Maximum single travel	Distance	Longest travel	279.60 km	40.66
Average depth	Sea floor depth	Mean sea depth along the travel	−129.60 m	14.22
Maximum depth	Sea floor depth	Maximum depth along the travel	−640.21 m	61.20
Prop. travel_‒150_	Sea floor depth	Proportion of the travel with sea depth < −150 m	0.84	0.02

Note

The mean values and standard error (*SE*) were obtained for all species and for the models M1, M2, and M3 (Total travel‐Prop. travel_−150_).

### 2.3 Species data

We constructed a phylogenetic tree using a synthesis‐based phylogeny generated from the TimeTree database (Kumar et al., 2017). TimeTree generates an optimal phylogenetic tree compared with other candidate topologies (Hedges et al., 2015). This approach is suitable for testing evolutionary hypotheses, and it usually produces similar results to those generated by purposely constructed phylogenies (Li et al., 2019). The pairwise divergence times between species were used to calculate the 10th percentile of the distances (measured in Mya), evaluating the species phylogenetic isolation relative to the complete pool of insular species (a measure related to the interspecific niche overlap; Münkemüller et al., 2014). This distance will be greater if the species shows a distant phylogenetic relationship relative to other island species, which could favor the colonization potential of this species. The species were also grouped in the following categories: snakes/lizards, island endemic/mainland, and based on trophic preferences (vertebrates/invertebrates). We also included the average total length (snout tip to the tip of the tail for adult specimens) for each species. These data were obtained from several previously published sources (Baier et al., 2013; Di Nicola & Mezzadri, 2018; Geniez, 2015; Schleich et al., 1996; Speybroeck et al., 2016).

### 2.4 Data analysis

The analyses tested (i) the strength of the phylogenetic signal in the dispersal patterns, and (ii) the effects of the species groups (i.e., phylogenetic isolation, snakes, endemics, trophic rank, and body size) in the dispersal patterns. Continuous variables with skew and kurtosis values that indicated non‐normality were logarithmically transformed prior to the analyses (Sokal & Rohlf, 1995). The species associations with the variables describing the components of the dispersal paths were visualized using principal component analysis (PCA; Pearson, 1901).

The intensity of the phylogenetic signal was determined by calculating Pagel's lambda and Blomberg's K statistics (Münkemüller et al., 2012). Pagel's lambda values vary between 0 and 1, where 0 denotes that a trait has evolved independently of the phylogeny and values close to 1 correspond to a Brownian model that indicates a phylogenetic signal during trait evolution (Pagel, 1999). Blomberg's K values vary between 0 and ∞, where values of K < 1 represent less phylogenetic signal than that expected under Brownian motion (Blomberg et al., 2003). When estimating the phylogenetic signal, we considered the uncertainty of the cost surface models and the differences between groups of squamates (if these groups included more than 30 species because the lambda and K parameters are sensitive to small phylogenies; Münkemüller et al., 2012). These analyses were conducted using the sensiPhy package (Paterno et al., 2018) for the R environment (R Development Core Team, 2021).

Associations between the species groups and characteristics of the dispersal paths were tested using phylogenetic linear regression models (PLMs; Revell, 2010). Total length was included in the models as an interacting term with snake/lizard category because most of the lizards in the region have total length values in the lower range for snakes (Speybroeck et al., 2016). PLMs were built by incorporating several phylogenetic structures for the error term: Brownian model (BM), the Ornstein–Uhlenbeck model with fixed root (OU1), the Ornstein–Uhlenbeck model with random root (OU2), Pagel's lambda, Pagel's kappa, Pagel's delta, and the early burst model (EB; Ho & Ané, 2014; Pagel, 1999). In these models, we also accounted for spatial effects (e.g., those associated with the uneven distribution of islands throughout the basin), including a variable generated with the centroids of the geographical coordinates of species. The optimal PLM was selected after comparing them against a null model (without covariance structures) using the delta Akaike's information criterion (AIC) and AIC weights (Burnham & Anderson, 2002). Models with delta AIC < 2 had great support, and AIC weights close to 1 indicated a higher probability of being the best candidate (Symonds & Moussalli, 2011). These analyses were conducted with the phylolm package (Ho & Ané, 2014) for the R environment.

## 3 RESULTS

**FIGURE 3:**
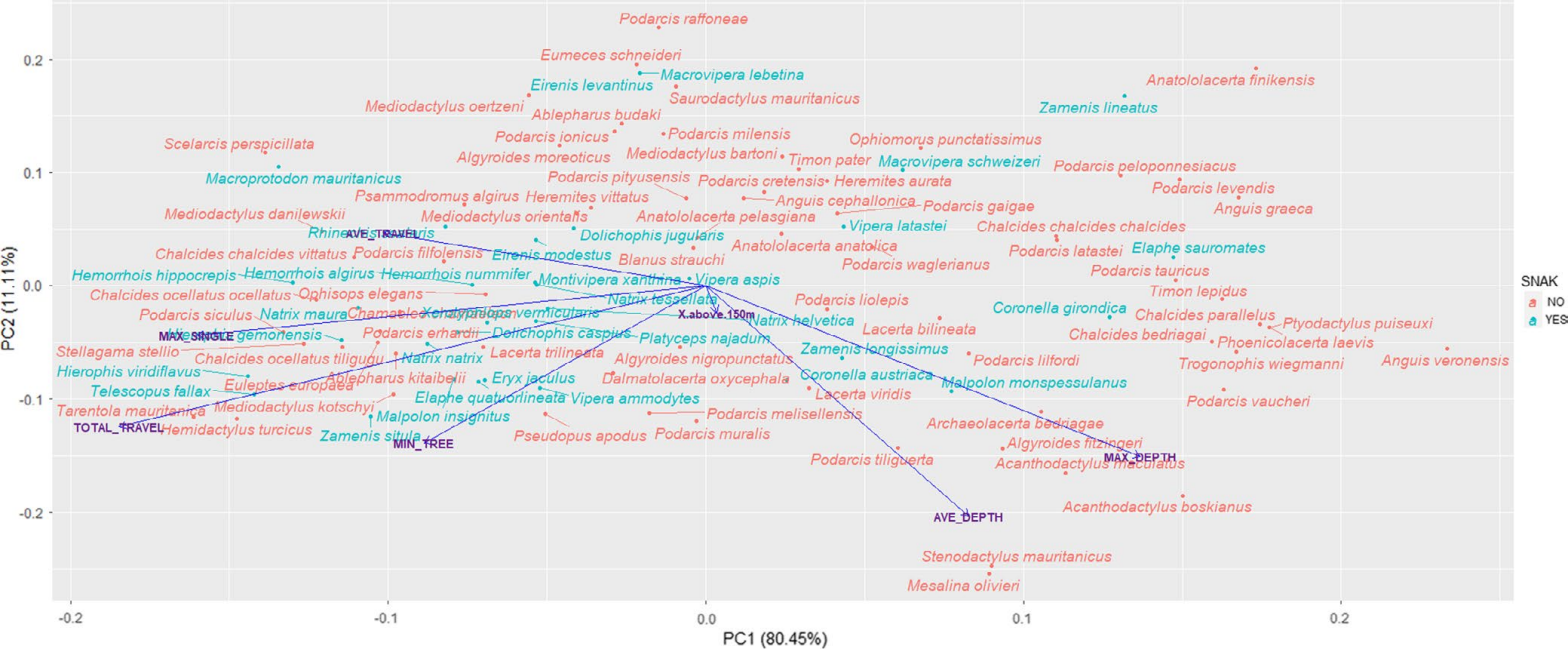
PCA scatter plot showing the variation in the characteristics of the paths (blue vectors) among species of insular squamates. Lizards, red; Snakes, blue

The first two axes obtained by PCA accounted for a large proportion of the variance (cumulative proportion = 0.916, PC1 = 0.805, PC2 = 0.111; Figure 3). On the first axis, the variables that described the distance traveled (total distance traveled, average distance traveled, and maximum distance traveled in a single trip) accounted for 67.7% of the total variance (Figure 3). On the second axis, the variables that described the bypassed sea depth (average depth and maximum depth along the routes) accounted for 61.9% of the total variance (Figure 3).

**FIGURE 4:**
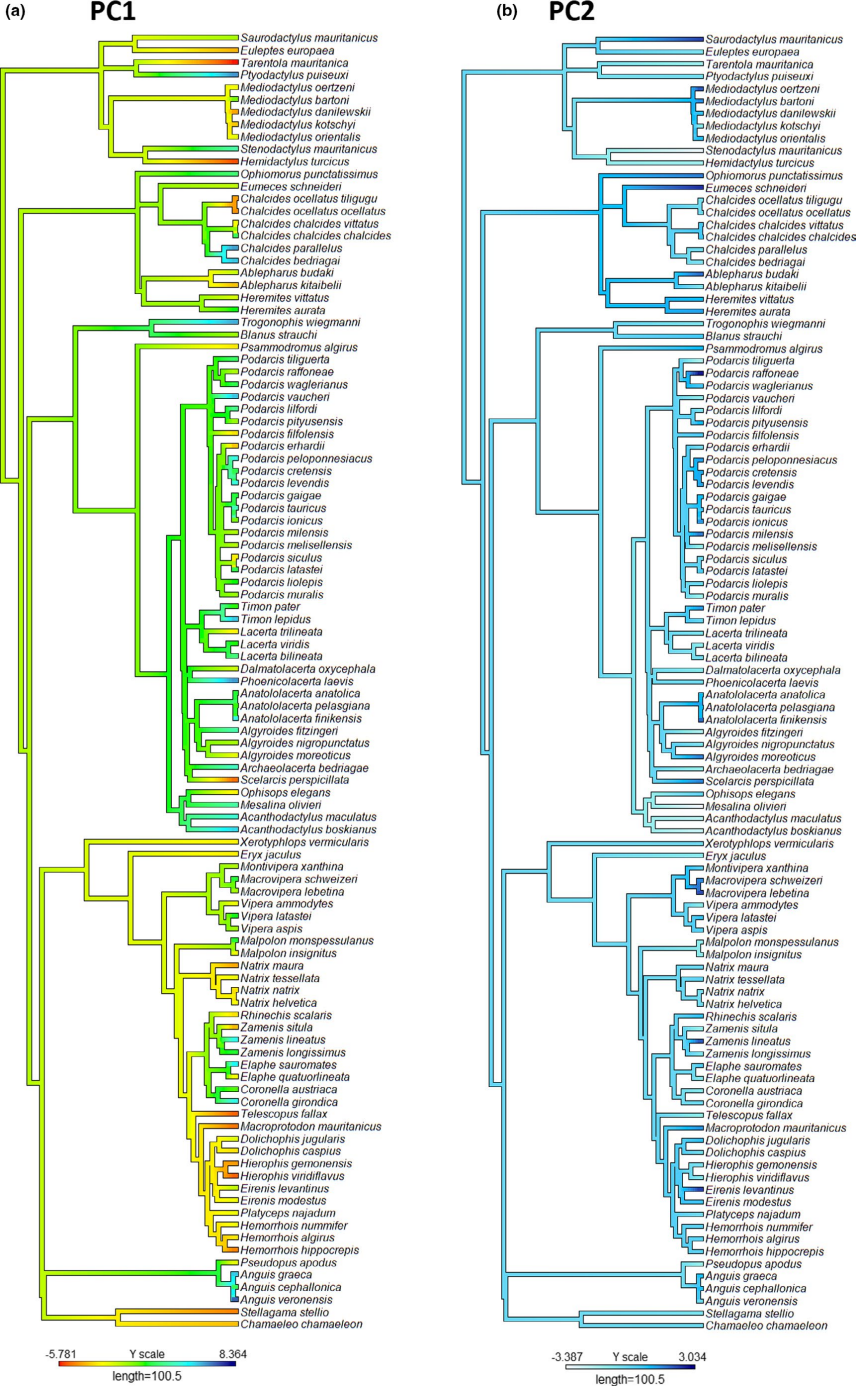
Squamata species (Mediterranean islands) phylogenetic tree, showing the mapped values of the first two PCA axes (a) PC1 (travel distance); (b) PC2 (travel depth)

The phylogenetic relationships among species on the first two axes obtained by PCA are shown in Figure 4. The estimates of Pagel's lambda and Blomberg's K indicated that no phylogenetic signal was present in any of the path properties (Figure 4; Table 2). In all cases, the phylogenetic signal was either insignificant or significant and close to 0 (i.e., phylogenetically related species shared fewer similarities in terms of their dispersal with each other than to those at greater evolutionary distances; Table 2). We also detected no phylogenetic signal when lizards and snakes were evaluated separately (Table 3).

**TABLE 2:** Evaluation of the phylogenetic signal (Pagel's λ and Blomberg's K) for the dispersion variables, including the uncertainty generated by the three dispersion models (M1–M3) for the variables total travel to prop. travel_−150_

		λ	K
Minimum tree	Estimate	0.209	0.089
	*p*‐Value	.0079	.0139
Total travel	Estimate	0.214	0.063
	Estimate 95% CI	0.153‒0.276	0.057‒0.069
	*p*‐Value	.4663	.5044
Average travel	Estimate	0.008	0.081
	Estimate 95% CI	0.004‒0.011	0.074‒0.087
	*p*‐Value	.8828	.4496
Max single travel	Estimate	0.069	0.056
	Estimate 95% CI	0.053‒0.084	0.053‒0.060
	*p*‐Value	.4404	.5182
Average depth	Estimate	0.003	0.162
	Estimate 95% CI	0.0006‒0.0044	0.150‒0.174
	*p*‐Value	.9607	.1938
Max depth	Estimate	0.012	0.066
	Estimate 95% CI	0.00‒0.028	0.058‒0.074
	*p*‐Value	.9347	.5419
Prop. travel_‒150_	Estimate	0.137	0.059
	Estimate 95% CI	0.116‒0.157	0.058‒0.061
	*p*‐Value	.3202	.3389

**TABLE 3:** Evaluation of the phylogenetic signal (Pagel's λ and Blomberg's K) for the dispersion variables, separating the groups of Squamata snakes and lizards

	λ	*p*‐Value	K	*p*‐Value
Minimum tree				
Snakes	0.00007	1.00	0.083	.331
Lizards	0.00005	1.00	0.188	.043
Total travel				
Snakes	0.00005	1.00	0.160	.509
Lizards	0.019	.855	0.039	.652
Average travel				
Snakes	0.00004	1.00	0.127	.732
Lizards	0.064	.360	0.035	.829
Max single travel				
Snakes	0.00006	1.00	0.187	.341
Lizards	0.053	.525	0.037	.763
Average depth				
Snakes	0.00005	1.00	0.179	.366
Lizards	0.577	.683	0.094	.017
Max depth				
Snakes	0.080	.488	0.151	.585
Lizards	0.576	.138	0.061	.177
Prop. travel_−150_				
Snakes	0.590	.350	0.196	.286
Lizards	0.144	.171	0.052	.323

**FIGURE 5:**
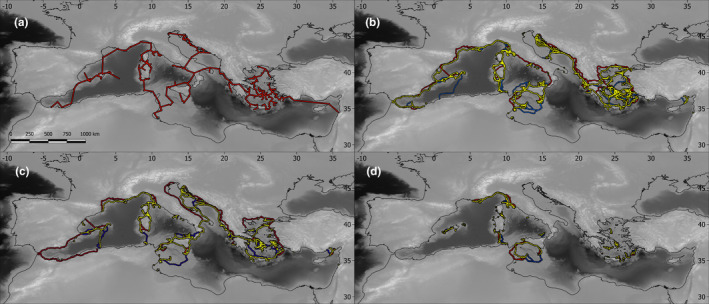
Dispersal paths of Squamata in the Mediterranean islands. (a) Minimum shortest distance; (b) modeled paths in lizards; (c) snakes; (d) island endemics. Blue, M1; yellow, M2; red, M3. When the paths overlap, only M2 is shown

The model with no phylogenetic covariance structures obtained the best fit for this data set (Table 4). The PLMs showed that the bypassed sea depth had a significant spatial component (average depth, maximum depth, and proportion of trip above −150 m; Table 5), where they indicated a significant negative association with longitude. This result implied that the dispersal paths in the eastern Mediterranean bypassed deeper marine regions (Figure 5). The regression analyses also showed that snakes traveled larger average distances whereas island endemics traveled shorter average distances and that small‐sized lizards traveled larger distances than other groups of squamates (Table 5).

**TABLE 4:** Candidate phylogenetic linear regression models evaluating the association among dispersion variables and Squamata traits/groups, including several phylogenetic covariance structures or none (null model, Null)

	BM	OU1	OU2	Lambda	Kappa	Delta	EB	Null
Minimum tree								
ΔAIC	91.62	0.96	0.96	2.00	31.60	73.09	93.62	0.00
AICw	0.00	0.24	0.24	0.14	0.00	0.00	0.00	0.38
Total travel								
ΔAIC	141.40	9.34	9.34	2.00	43.62	120.43	143.40	0.00
AICw	0.00	0.007	0.007	0.26	0.00	0.00	0.00	0.72
Average travel								
ΔAIC	157.92	12.67	12.67	2.00	48.38	135.25	159.92	0.00
AICw	0.00	0.001	0.001	0.27	0.00	0.00	0.00	0.73
Max single travel								
ΔAIC	136.82	9.33	9.33	2.00	44.11	115.83	138.82	0.00
AICw	0.00	0.007	0.007	0.26	0.00	0.00	0.00	0.72
Average depth								
ΔAIC	48.41	3.46	3.46	2.16	27.89	35.59	50.41	0.00
AICw	0.00	0.11	0.11	0.19	0.00	0.00	0.00	0.59
Max depth								
ΔAIC	91.69	8.10	8.10	2.00	39.73	76.74	93.69	0.00
AICw	0.00	0.01	0.01	0.26	0.00	0.00	0.00	0.72
Prop. travel_−150_								
ΔAIC	132.48	9.35	9.35	2.00	49.40	111.28	134.48	0.00
AICw	0.00	0.007	0.007	0.26	0.00	0.00	0.00	0.72

Note

The best candidate is the one that shows a delta AIC (ΔAIC) <2 and AIC weight (AICw) close to 1.

Abbreviations: BM, Brownian motion model; delta, Pagel's δ model; EB, early burst model; kappa, Pagel's κ model; lambda, Pagel's λ model; OU1, Ornstein–Uhlenbeck model with fixed root; OU2, Ornstein–Uhlenbeck model with random root.

**TABLE 5:** Best phylogenetic linear regression model generated by AIC selection, evaluating the association between predictor variables and travel descriptors

	Model statistics		Variables	Estimate	*p*‐Value
Minimum tree	*R* ^2^	.209	Latitude	0.147	.0003
	AIC	333.127	Endemic	−0.622	.0524
	AICw	0.297	TL:no	−0.524	.0353
Total travel	*R* ^2^	.067	TL:no	−0.580	.1557
	AIC	452.252			
	AICw	0.315			
Average travel	*R* ^2^	.150	Latitude	−0.101	.0439
	AIC	346.821	Snake	0.596	.0282
	AICw	0.511	Endemic	−0.850	.0129
Max single travel	*R* ^2^	.062	TL:no	−0.554	.1200
	AIC	423.253			
	AICw	0.377			
Average depth	*R* ^2^	.144	Longitude	−0.041	.0005
	AIC	327.907	Snake	−0.399	.0933
	AICw	0.467			
Max depth	*R* ^2^	.112	Longitude	−0.047	.0035
	AIC	394.572	Snake	−0.719	.0287
	AICw	0.572			
Prop. travel_−150_	*R* ^2^	.236	Latitude	0.029	.0023
	AIC	−13.733	Longitude	−0.007	.0044
	AICw	0.373	Endemic	−0.129	.0296

Abbreviations: AIC, Akaike information criterion; AICw, AIC weight; TL :no, interaction between total length and snake category (yes/no).

## 4 DISCUSSION

In this study, we modeled the dispersal paths of squamates throughout the Mediterranean archipelagos. The dispersal models accounted for the uncertainty regarding the resistance imposed by the sea on the movements of species, which could generate a range of routes, particularly when evaluating long traveling distances. However, the analyses obtained similar results when the distances traveled were estimated without assuming a dispersal direction and landscape resistance. Our results highlighted the lack of a phylogenetic signal in the dispersal paths when evaluating both the distances traveled and bypassed sea depth. This finding indicated that no squamate lineages in the Mediterranean basin had greater dispersal capacities than others. It was also feasible that more intense interspecific competition between phylogenetic relatives in small islands (Escoriza, 2020) could counteract the effects of traits that might possibly enhance transmarine dispersal.

Snakes and lizards differed in terms of their dispersal patterns. The analyses revealed that snakes show greater average traveled distances. Thus, when snakes traveled similar distances to lizards, they did so with fewer “stops” (i.e., by populating less intermediate islands). The lower number of stops may have been a consequence of the failure of snakes to colonize small islets or to maintain stable populations on these islets for long periods because most of the snakes, even those with small body sizes, occupied higher trophic levels than lizards (Pernetta et al., 2011). However, this result could also have been a human‐induced artifact given that the longest paths traveled by Mediterranean snakes are due to translocations (e.g., *Erix jaculus*, *Hierophis viridiflavus,* or *Hemorrhois hippocrepis*; Utiger & Schätti, 2004; Pinya & Carretero, 2011; Insacco et al., 2015), and thus, they do not follow the expected sequence of island chains. Natricine snakes are possible exceptions because they are efficient marine dispersers (Brischoux & Kornilev, 2014; Kyriazi et al., 2013) but confined to large islands due to their requirements for permanent freshwater habitats (Zotos et al., 2021). However, in this group of semi‐aquatic snakes, at least two island populations of *Natrix maura* (Mallorca and Sardinia) were introduced (Guicking et al., 2008).

Our analyses also indicated that the island endemics had shorter average traveled distances. Thus, when endemic species traveled similar distances to mainland species, they had a greater number of “stops” (or by populating a larger number of intermediate islands). This is consequence of the process of radial dispersion by following an ordered array of islands (i.e., from the closest to the furthest). This is to be expected considering that very few populations of endemic squamates had anthropogenic origins (Lo Cascio et al., 2006; Pérez‐Mellado et al., 2017). The regression models demonstrated the importance of the spatial component in the dispersal patterns. For example, squamates crossed deeper regions of the sea in the eastern Mediterranean region where the density of islands is also greater (Arnold, 2008). This finding could be a consequence of greater success on the transmarine routes in this region, which is highly dependent on stochastic factors (Simpson, 1940).

Our analyses indicated that a morphological trait could influence the transmarine dispersal process, at least in lizards. Body size was negatively associated with the distance traveled in lizards, possibly because small lizards can more readily populate tiny intermediate islands (Delaugerre & Corti, 2020; Pafilis et al., 2020) or be transported accidentally by man (Austin, 1999). In addition, small‐bodied lizards tend to have larger population sizes, which could favor the probability of successful dispersal by rafting, including the use of a wider range of raft sizes (Hsu et al., 2021; Novosolov et al., 2016). Examples of highly successful island colonizers with small body sizes are found in several distantly related lizard lineages within the Mediterranean region, including skinks (e.g., *Ablepharus*), geckoes (*Euleptes* and *Mediodactylus*), and lacertids (*Ophisops*), and thus, a phylogenetic signal was not detected.

## 5 CONCLUSIONS

In this study, for the first time, we evaluated the dispersal patterns of Squamata in the Mediterranean islands by estimating several parameters to quantify the difficulty of reaching these islands. The results highlighted the lack of any phylogenetic signal in the dispersal process, thereby indicating that no evolutionary lineages had superior colonization capacities. Our analyses also revealed important differences in the dispersal process for snakes compared with lizards, although these differences could have been human‐induced artifacts because some long‐distance dispersals of snakes may possibly have been due to accidental introductions. In lizards, small body size possibly enhanced the probability of success over long‐distance sea routes.

## CONFLICT OF INTEREST

None declared.

## AUTHOR CONTRIBUTION

**Daniel Escoriza:** Conceptualization (equal); Data curation (equal); Formal analysis (equal); Funding acquisition (equal); Investigation (equal); Methodology (equal); Project administration (equal).

## Supporting information

Appendices S1 and S2

## Data Availability

The raw data used in the present study are available within the article or its Supporting Information. Dataset for modeled trajectories is available at Dryad Digital Repository: https://doi.org/10.5061/dryad.zw3r2288c
