# Supplementary material for: Dispersal patterns of Squamata in the Mediterranean: An evolutionary perspective
Source: Ecol Evol. 2021 Sep 24;11(21):14733–43. doi: 10.1002/ece3.8159 (PMC8571612; doi:10.1002/ece3.8159)
Supplement: Supplementary file 1 — Appendices S1 and S2 [file ECE3-11-14733-s001.docx]

Appendix S1. List of species included in the study, along with the main references used to delimit their distribution.

| Species | Main reference |
| --- | --- |
| *Ablepharus budaki* | Kalaentzis, K., Strachinis, I., Katsiyiannis, P., Oefinger, P. and Kazilas, C., 2018. New records and an updated list of the herpetofauna of Kastellorizo and the adjacent islet Psomi Dodecanese, Greece. Herpetology Notes, 11, pp.1009-1019. |
| *Ablepharus kitaibelii* | Chondropoulos, B.P., 1986. A checklist of the Greek reptiles. I. The lizards. Amphibia-reptilia, 73, pp.217-235. |
| *Acanthodactylus boskianus* | Nouira S., 2004. Biodiversité et statut écologique des scorpions et reptiles des îles Kneiss. Projet de préservation de la biodiversité dans la Réserve Naturelle des Iles Kneiss. GEF-UNDP, 8 pp. unpublished document, available at http://www.initiative-pim.org/en/node/47184 |
| *Acanthodactylus maculatus* | Corti C., 2015. L’Herpétofaune des Îles Kerkennah. Compte-rendu de prospections. Initiative PIM.66 p. Collaboration : Said Nouira. |
| *Algyroides fitzingeri* | Delaugerre, M., & Cheylan, M. 1992. Atlas de Repartition des Batraciens et Reptiles de Corse. Montpellier: Parc Naturel Regional de Corse-Ecole Pratique des Hautes Études. |
| *Algyroides moreoticus* | Chondropoulos, B.P., 1986. A checklist of the Greek reptiles. I. The lizards. Amphibia-reptilia, 73, pp.217-235. |
| *Algyroides nigropunctatus* | Chondropoulos, B.P., 1986. A checklist of the Greek reptiles. I. The lizards. Amphibia-reptilia, 73, pp.217-235. |
| *Anatololacerta anatolica* | Chondropoulos, B.P., 1986. A checklist of the Greek reptiles. I. The lizards. Amphibia-reptilia, 73, pp.217-235. |
| *Anatololacerta finikensis* | Chondropoulos, B.P., 1986. A checklist of the Greek reptiles. I. The lizards. Amphibia-reptilia, 73, pp.217-235. |
| *Anatololacerta pelasgiana* | Chondropoulos, B.P., 1986. A checklist of the Greek reptiles. I. The lizards. Amphibia-reptilia, 73, pp.217-235. |
| *Anguis cephallonica* | Chondropoulos, B.P., 1986. A checklist of the Greek reptiles. I. The lizards. Amphibia-reptilia, 73, pp.217-235. |
| *Anguis graeca* | Chondropoulos, B.P., 1986. A checklist of the Greek reptiles. I. The lizards. Amphibia-reptilia, 73, pp.217-235. |
| *Anguis veronensis* | Renet, J., Lucente, D., Delaugerre, M., Gerriet, O., Deso, G., Abbattista, C. and Cimmaruta, R., 2018. Discovery of an Italian slow worm Anguis veronensis Pollini, 1818 population on a Western Mediterranean Island confirmed by genetic analysis. Acta Herpetologica, 132, pp.165-169. |
| *Archaeolacerta bedriagae* | Delaugerre, M., & Cheylan, M. 1992. Atlas de Repartition des Batraciens et Reptiles de Corse. Montpellier: Parc Naturel Regional de Corse-Ecole Pratique des Hautes Études. |
| *Blanus strauchi* | Chondropoulos, B.P., 1986. A checklist of the Greek reptiles. I. The lizards. Amphibia-reptilia, 73, pp.217-235. |
| *Chalcides bedriagai* | Pleguezuelos, J. M., Márquez, R., & Lizana, M., 2004. Atlas y libro rojo de los anfibios y reptiles de España. Madrid: Organismo Autónomo de Parques Nacionales. |
| *Chalcides chalcides chalcides* | Sindaco, R., Doria, G., Razzetti, E., & Bernini, F. 2006. Atlante degli Anfibi e Rettili d’Italia. Firenze: Polistampa |
| *Chalcides chalcides vittatus* | Sindaco, R., Doria, G., Razzetti, E., & Bernini, F. 2006. Atlante degli Anfibi e Rettili d’Italia. Firenze: Polistampa |
| *Chalcides ocellatus ocellatus* | Chondropoulos, B.P., 1986. A checklist of the Greek reptiles. I. The lizards. Amphibia-reptilia, 73, pp.217-235. |
| *Chalcides ocellatus tiligugu* | Sindaco, R., Doria, G., Razzetti, E., & Bernini, F. 2006. Atlante degli Anfibi e Rettili d’Italia. Firenze: Polistampa |
| *Chalcides parallelus* | Pleguezuelos, J. M., Márquez, R., & Lizana, M., 2004. Atlas y libro rojo de los anfibios y reptiles de España. Madrid: Organismo Autónomo de Parques Nacionales. |
| *Chamaeleo chamaeleon* | Chondropoulos, B.P., 1986. A checklist of the Greek reptiles. I. The lizards. Amphibia-reptilia, 73, pp.217-235. |
| *Coronella austriaca* | Sindaco, R., Doria, G., Razzetti, E., & Bernini, F. 2006. Atlante degli Anfibi e Rettili d’Italia. Firenze: Polistampa |
| *Coronella girondica* | Pleguezuelos, J. M., Márquez, R., & Lizana, M., 2004. Atlas y libro rojo de los anfibios y reptiles de España. Madrid: Organismo Autónomo de Parques Nacionales. |
| *Dalmatolacerta oxycephala* | Krystufek, B. and Kletecki, E., 2007. Biogeography of small terrestrial vertebrates on the Adriatic landbridge islands. Folia zoologica, 563, p.225. |
| *Dolichophis caspius* | Chondropoulos, B. P., 1989. A checklist of the Greek reptiles. I. The snakes. Herpetozoa, 2, 3-36. |
| *Dolichophis jugularis* | Chondropoulos, B. P., 1989. A checklist of the Greek reptiles. I. The snakes. Herpetozoa, 2, 3-36. |
| *Eirenis levantinus* | Baier, F., Sparrow, D.J. and Wiedl, H.J., 2013. The amphibians and reptiles of Cyprus. Frankfurt am Main: Edition Chimaira. |
| *Eirenis modestus* | Chondropoulos, B. P., 1989. A checklist of the Greek reptiles. I. The snakes. Herpetozoa, 2, 3-36. |
| *Elaphe quatuorlineata* | Chondropoulos, B. P., 1989. A checklist of the Greek reptiles. I. The snakes. Herpetozoa, 2, 3-36. |
| *Elaphe sauromates* | Chondropoulos, B. P., 1989. A checklist of the Greek reptiles. I. The snakes. Herpetozoa, 2, 3-36. |
| *Eryx jaculus* | Chondropoulos, B. P., 1989. A checklist of the Greek reptiles. I. The snakes. Herpetozoa, 2, 3-36. |
| *Euleptes europaea* | Delaugerre, M., & Cheylan, M. 1992. Atlas de Repartition des Batraciens et Reptiles de Corse. Montpellier: Parc Naturel Regional de Corse-Ecole Pratique des Hautes Études. |
| *Eumeces schneiderii* | Baier, F., Sparrow, D.J. and Wiedl, H.J., 2013. The amphibians and reptiles of Cyprus. Frankfurt am Main: Edition Chimaira. |
| *Hemidactylus turcicus* | Chondropoulos, B.P., 1986. A checklist of the Greek reptiles. I. The lizards. Amphibia-reptilia, 73, pp.217-235. |
| *Hemorrhois algirus* | Borg, M.J., & Schembri, P.J., 1991. A short note of the herpetofauna of Gozo. British Herpetological Society Bulletin, 38, 8-9. |
| *Hemorrhois hippocrepis* | Sindaco, R., Doria, G., Razzetti, E., & Bernini, F. 2006. Atlante degli Anfibi e Rettili d’Italia. Firenze: Polistampa |
| *Hemorrhois nummifer* | Chondropoulos, B. P., 1989. A checklist of the Greek reptiles. I. The snakes. Herpetozoa, 2, 3-36. |
| *Heremites auratus* | Chondropoulos, B.P., 1986. A checklist of the Greek reptiles. I. The lizards. Amphibia-reptilia, 73, pp.217-235. |
| *Heremites vittatus* | Corti C., 2015. L’Herpétofaune des Îles Kerkennah. Compte-rendu de prospections. Initiative PIM.66 p. Collaboration : Said Nouira. |
| *Hierophis gemonensis* | Chondropoulos, B. P., 1989. A checklist of the Greek reptiles. I. The snakes. Herpetozoa, 2, 3-36. |
| *Hierophis viridiflavus* | Delaugerre, M., & Cheylan, M. 1992. Atlas de Repartition des Batraciens et Reptiles de Corse. Montpellier: Parc Naturel Regional de Corse-Ecole Pratique des Hautes Études. |
| *Lacerta bilineata* | Sindaco, R., Doria, G., Razzetti, E., & Bernini, F. 2006. Atlante degli Anfibi e Rettili d’Italia. Firenze: Polistampa |
| *Lacerta trilineata* | Chondropoulos, B.P., 1986. A checklist of the Greek reptiles. I. The lizards. Amphibia-reptilia, 73, pp.217-235. |
| *Lacerta viridis* | Chondropoulos, B.P., 1986. A checklist of the Greek reptiles. I. The lizards. Amphibia-reptilia, 73, pp.217-235. |
| *Macroprotodon mauritanicus* | Mayol, J., 1997. Biogeografía de los Anfibios y Reptiles de las Islas Baleares. Distribución y Biogeografía de los Anfibios y Reptiles en España y Portugal, 10, 371-379. |
| *Macrovipera lebetinus* | Baier, F., Sparrow, D.J. and Wiedl, H.J., 2013. The amphibians and reptiles of Cyprus. Frankfurt am Main: Edition Chimaira. |
| *Macrovipera schweizeri* | Chondropoulos, B. P., 1989. A checklist of the Greek reptiles. I. The snakes. Herpetozoa, 2, 3-36. |
| *Malpolon insignitus* | Chondropoulos, B. P., 1989. A checklist of the Greek reptiles. I. The snakes. Herpetozoa, 2, 3-36. |
| *Malpolon monspessulanus* | Cheylan, M., 1983. Statut actuel des reptiles et amphibiens de l'archipel des iles d'Hyères (Var, sud-est de la France). Travaux scientifiques du Parc national de Port-Cros, 9, 35-51. |
| *Mediodactylus bartoni* | Kotsakiozi, P., Jablonski, D., Ilgaz, Ç., Kumlutas, Y., Avci, A., Meiri, S., Itescu, Y., Kukushkin, O., Gvoždík, V., Scillitani, G., & Roussos, S. A., 2018. Multilocus phylogeny and coalescent species delimitation in Kotschy's gecko, Mediodactylus kotschyi: Hidden diversity and cryptic species. Molecular Phylogenetics and Evolution, 125, 177-187. |
| *Mediodactylus danilewskii* | Kotsakiozi, P., Jablonski, D., Ilgaz, Ç., Kumlutas, Y., Avci, A., Meiri, S., Itescu, Y., Kukushkin, O., Gvoždík, V., Scillitani, G., & Roussos, S. A., 2018. Multilocus phylogeny and coalescent species delimitation in Kotschy's gecko, Mediodactylus kotschyi: Hidden diversity and cryptic species. Molecular Phylogenetics and Evolution, 125, 177-187. |
| *Mediodactylus kotschyi* | Kotsakiozi, P., Jablonski, D., Ilgaz, Ç., Kumlutas, Y., Avci, A., Meiri, S., Itescu, Y., Kukushkin, O., Gvoždík, V., Scillitani, G., & Roussos, S. A., 2018. Multilocus phylogeny and coalescent species delimitation in Kotschy's gecko, Mediodactylus kotschyi: Hidden diversity and cryptic species. Molecular Phylogenetics and Evolution, 125, 177-187. |
| *Mediodactylus oertzeni* | Kotsakiozi, P., Jablonski, D., Ilgaz, Ç., Kumlutas, Y., Avci, A., Meiri, S., Itescu, Y., Kukushkin, O., Gvoždík, V., Scillitani, G., & Roussos, S. A., 2018. Multilocus phylogeny and coalescent species delimitation in Kotschy's gecko, Mediodactylus kotschyi: Hidden diversity and cryptic species. Molecular Phylogenetics and Evolution, 125, 177-187. |
| *Mediodactylus orientalis* | Kotsakiozi, P., Jablonski, D., Ilgaz, Ç., Kumlutas, Y., Avci, A., Meiri, S., Itescu, Y., Kukushkin, O., Gvoždík, V., Scillitani, G., & Roussos, S. A., 2018. Multilocus phylogeny and coalescent species delimitation in Kotschy's gecko, Mediodactylus kotschyi: Hidden diversity and cryptic species. Molecular Phylogenetics and Evolution, 125, 177-187. |
| *Mesalina olivieri* | Corti C., 2015. L’Herpétofaune des Îles Kerkennah. Compte-rendu de prospections. Initiative PIM.66 p. Collaboration : Said Nouira. |
| *Montivipera xanthina* | Chondropoulos, B. P. 1989. A checklist of the Greek reptiles. I. The snakes. Herpetozoa, 2, 3-36. |
| *Natrix helvetica* | Sindaco, R., Doria, G., Razzetti, E., & Bernini, F. 2006. Atlante degli Anfibi e Rettili d’Italia. Firenze: Polistampa |
| *Natrix maura* | Sindaco, R., Doria, G., Razzetti, E., & Bernini, F. 2006. Atlante degli Anfibi e Rettili d’Italia. Firenze: Polistampa |
| *Natrix natrix* | Chondropoulos, B. P., 1989. A checklist of the Greek reptiles. I. The snakes. Herpetozoa, 2, 3-36. |
| *Natrix tessellata* | Chondropoulos, B. P., 1989. A checklist of the Greek reptiles. I. The snakes. Herpetozoa, 2, 3-36. |
| *Ophiomorus punctatissimus* | Chondropoulos, B.P., 1986. A checklist of the Greek reptiles. I. The lizards. Amphibia-reptilia, 73, pp.217-235. |
| *Ophisops elegans* | Chondropoulos, B.P., 1986. A checklist of the Greek reptiles. I. The lizards. Amphibia-reptilia, 73, pp.217-235. |
| *Phoenicolacerta laevis* | Tohmé, G., Tohmé, H., Abi-Saeed, M., Ramadan-Jaradi, G., Hraoui-Bloquet, S., & Merheb, B., 2004. Biodiversity assessment and monitoring in the protected areas/Lebanon LEB/95/G31: Palm Islands Nature Reserve. Final Report. Beirut: Ministry of Environment-Lebanese University. |
| *Platyceps najadum* | Chondropoulos, B. P., 1989. A checklist of the Greek reptiles. I. The snakes. Herpetozoa, 2, 3-36. |
| *Podarcis cretensis* | Spilani, L., Bougiouri, K., Antoniou, A., Psonis, N., Poursanidis, D., Lymberakis, P. and Poulakakis, N., 2019. Multigene phylogeny, phylogeography and population structure of Podarcis cretensis species group in south Balkans. Molecular Phylogenetics and Evolution, 138, pp.193-204. |
| *Podarcis erhardii* | Chondropoulos, B.P., 1986. A checklist of the Greek reptiles. I. The lizards. Amphibia-reptilia, 73, pp.217-235. |
| *Podarcis filfolensis* | Sindaco, R., Doria, G., Razzetti, E., & Bernini, F. 2006. Atlante degli Anfibi e Rettili d’Italia. Firenze: Polistampa |
| *Podarcis gaigae* | Itescu, Y., Schwarz, R., Donihue, C.M., Slavenko, A., Roussos, S.A., Sagonas, K., Valakos, E.D., Foufopoulos, J., Pafilis, P. and Meiri, S., 2018. Inconsistent patterns of body size evolution in co-occurring island reptiles. Global Ecology and Biogeography, 27(5), pp.538-550. |
| *Podarcis ionicus* | Chondropoulos, B.P., 1986. A checklist of the Greek reptiles. I. The lizards. Amphibia-reptilia, 73, pp.217-235. |
| *Podarcis latastei* | Senczuk, G., Castiglia, R. and Böhme, W., 2019. Podarcis siculus latastei (Bedriaga, 1879) of the Western Pontine Islands (Italy) raised to the species rank, and a brief taxonomic overview of Podarcis lizards. Acta Herpetologica, 14(2), pp.71-80. |
| *Podarcis levendis* | Lymberakis, P., Valakos, E., Sagonas, K. and Pafilis, P., 2016. The castaway: characteristic islet features affect the ecology of the most isolated European lizard. Acta Herpetologica, 11(2), pp.161-169. |
| *Podarcis lilfordi* | Mayol, J., 1997. Biogeografía de los Anfibios y Reptiles de las Islas Baleares. Distribución y Biogeografía de los Anfibios y Reptiles en España y Portugal, 10, 371-379. |
| *Podarcis liolepis* | Castilla, A. M., & Alí, M., 1997. Posibles cambios en la abundancia de reptiles en las islas Medes (Girona, Mediterráneo Occidental). Bolletí de la Societat d'Historia Natural de les Balears, 40, 163-168. |
| *Podarcis melisellensis* | Krystufek, B. and Kletecki, E., 2007. Biogeography of small terrestrial vertebrates on the Adriatic landbridge islands. Folia zoologica, 563, p.225. |
| *Podarcis milensis* | Chondropoulos, B.P., 1986. A checklist of the Greek reptiles. I. The lizards. Amphibia-reptilia, 73, pp.217-235. |
| *Podarcis muralis* | Sindaco, R., Doria, G., Razzetti, E., & Bernini, F. 2006. Atlante degli Anfibi e Rettili d’Italia. Firenze: Polistampa |
| *Podarcis peloponnesiacus* | Chondropoulos, B.P., 1986. A checklist of the Greek reptiles. I. The lizards. Amphibia-reptilia, 73, pp.217-235. |
| *Podarcis pityusensis* | Mayol, J., 1997. Biogeografía de los Anfibios y Reptiles de las Islas Baleares. Distribución y Biogeografía de los Anfibios y Reptiles en España y Portugal, 10, 371-379. |
| *Podarcis raffonei* | Sindaco, R., Doria, G., Razzetti, E., & Bernini, F. 2006. Atlante degli Anfibi e Rettili d’Italia. Firenze: Polistampa |
| *Podarcis siculus* | Sindaco, R., Doria, G., Razzetti, E., & Bernini, F. 2006. Atlante degli Anfibi e Rettili d’Italia. Firenze: Polistampa |
| *Podarcis tauricus* | Chondropoulos, B.P., 1986. A checklist of the Greek reptiles. I. The lizards. Amphibia-reptilia, 73, pp.217-235. |
| *Podarcis tiliguerta* | Delaugerre, M., & Cheylan, M. 1992. Atlas de Repartition des Batraciens et Reptiles de Corse. Montpellier: Parc Naturel Regional de Corse-Ecole Pratique des Hautes Études. |
| *Podarcis vaucheri* | Pleguezuelos, J. M., Márquez, R., & Lizana, M., 2004. Atlas y libro rojo de los anfibios y reptiles de España. Madrid: Organismo Autónomo de Parques Nacionales. |
| *Podarcis waglerianus* | Sindaco, R., Doria, G., Razzetti, E., & Bernini, F. 2006. Atlante degli Anfibi e Rettili d’Italia. Firenze: Polistampa |
| *Psammodromus algirus* | Sindaco, R., Doria, G., Razzetti, E., & Bernini, F. 2006. Atlante degli Anfibi e Rettili d’Italia. Firenze: Polistampa |
| *Pseudopus apodus* | Chondropoulos, B.P., 1986. A checklist of the Greek reptiles. I. The lizards. Amphibia-reptilia, 73, pp.217-235. |
| *Ptyodactylus puiseuxi* | Tohmé, G., Tohmé, H., Abi-Saeed, M., Ramadan-Jaradi, G., Hraoui-Bloquet, S., & Merheb, B., 2004. Biodiversity assessment and monitoring in the protected areas/Lebanon LEB/95/G31: Palm Islands Nature Reserve. Final Report. Beirut: Ministry of Environment-Lebanese University. |
| *Saurodactylus mauritanicus* | Pleguezuelos, J. M., Márquez, R., & Lizana, M., 2004. Atlas y libro rojo de los anfibios y reptiles de España. Madrid: Organismo Autónomo de Parques Nacionales. |
| *Scelarcis perspicillata* | Mayol, J., 1997. Biogeografía de los Anfibios y Reptiles de las Islas Baleares. Distribución y Biogeografía de los Anfibios y Reptiles en España y Portugal, 10, 371-379. |
| *Stellagama stellio* | Chondropoulos, B.P., 1986. A checklist of the Greek reptiles. I. The lizards. Amphibia-reptilia, 73, pp.217-235. |
| *Stenodactylus mauritanicus* | Corti C., 2015. L’Herpétofaune des Îles Kerkennah. Compte-rendu de prospections. Initiative PIM.66 p. Collaboration : Said Nouira. |
| *Tarentola mauritanica* | Sindaco, R., Doria, G., Razzetti, E., & Bernini, F. 2006. Atlante degli Anfibi e Rettili d’Italia. Firenze: Polistampa |
| *Telescopus fallax* | Chondropoulos, B. P., 1989. A checklist of the Greek reptiles. I. The snakes. Herpetozoa, 2, 3-36. |
| *Timon lepidus* | Cheylan, M., 2016. The extinction of the ocellated lizard Timon lepidus lepidus (Daudin, 1802) on the island of Porquerolles (Provence, France). Boletín de la Asociación Herpetológica Española, 27(2), pp.155-158. |
| *Timon pater* | Schleich, H. H., Kästle, W., & Kabisch, K., 1996. Amphibians and reptiles of North Africa. Koenigstein: Koeltz. |
| *Trogonophis wiegmanni* | Pleguezuelos, J. M., Márquez, R., & Lizana, M., 2004. Atlas y libro rojo de los anfibios y reptiles de España. Madrid: Organismo Autónomo de Parques Nacionales. |
| *Vipera ammodytes* | Chondropoulos, B. P., 1989. A checklist of the Greek reptiles. I. The snakes. Herpetozoa, 2, 3-36. |
| *Vipera aspis* | Sindaco, R., Doria, G., Razzetti, E., & Bernini, F. 2006. Atlante degli Anfibi e Rettili d’Italia. Firenze: Polistampa |
| *Vipera latastei* | Salvador, A., 2014. Fauna Ibérica: Reptiles. Madrid: MNCN-CSIC. |
| *Xerotyphlops vermicularis* | Chondropoulos, B. P., 1989. A checklist of the Greek reptiles. I. The snakes. Herpetozoa, 2, 3-36. |
| *Zamenis lineatus* | Sindaco, R., Doria, G., Razzetti, E., & Bernini, F. 2006. Atlante degli Anfibi e Rettili d’Italia. Firenze: Polistampa |
| *Zamenis longissimus* | Sindaco, R., Doria, G., Razzetti, E., & Bernini, F. 2006. Atlante degli Anfibi e Rettili d’Italia. Firenze: Polistampa |
| *Zamenis scalaris* | Mayol, J., 1997. Biogeografía de los Anfibios y Reptiles de las Islas Baleares. Distribución y Biogeografía de los Anfibios y Reptiles en España y Portugal, 10, 371-379. |
| *Zamenis situlus* | Chondropoulos, B. P., 1989. A checklist of the Greek reptiles. I. The snakes. Herpetozoa, 2, 3-36. |

Appendix S2. Mean value of the travel descriptors for each resistance model (M1, M2, M3) and species. M1.

| Species | Shortest path | Modeled distance M1 | Average distance M1 | Max distance M1 | Average depth M1 | Max depth M1 | % above -150m M1 |
| --- | --- | --- | --- | --- | --- | --- | --- |
| *Ablepharus budaki* | 2.9 | 1.1 | 0.6 | 1.1 | -473.1 | -772.6 | 0.1 |
| *Ablepharus kitaibelii* | 24.3 | 29.9 | 0.4 | 4.3 | -204.0 | -1103.1 | 0.6 |
| *Acanthodactylus boskianus* | 0.8 | 0.3 | 0.1 | 0.1 | -1.7 | -6.1 | 1.0 |
| *Acanthodactylus maculatus* | - | 0.4 | 0.4 | 0.4 | -2.7 | -4.0 | 1.0 |
| *Algyroides fitzingeri* | 4.9 | 0.6 | 0.1 | 0.2 | -7.2 | -36.3 | 1.0 |
| *Algyroides moreoticus* | 3.3 | 2.4 | 0.5 | 0.9 | -335.5 | -1288.6 | 0.6 |
| *Algyroides nigropunctatus* | 10.4 | 4.0 | 0.3 | 1.1 | -79.3 | -269.8 | 0.9 |
| *Anatololacerta anatolica* | 0.7 | 1.0 | 0.5 | 1.0 | -100.7 | -183.3 | 0.9 |
| *Anatololacerta finikensis* | 0.0 | 0.0 | 0.0 | 0.0 | -119.9 | -135.9 | 1.0 |
| *Anatololacerta pelasgiana* | 3.2 | 1.8 | 0.3 | 0.8 | -150.7 | -355.5 | 0.5 |
| *Anguis cephallonica* | 0.4 | 1.6 | 0.5 | 0.8 | -108.8 | -274.6 | 0.8 |
| *Anguis graeca* | - | 0.0 | 0.0 | 0.0 | -24.1 | -54.8 | 1.0 |
| *Anguis veronensis* | - | 0.0 | 0.0 | 0.0 | -4.0 | -5.1 | 1.0 |
| *Archaeolacerta bedriagae* | 1.1 | 0.5 | 0.1 | 0.2 | -6.4 | -36.3 | 1.0 |
| *Blanus strauchi* | 3.8 | 2.3 | 0.3 | 0.7 | -129.5 | -338.4 | 0.8 |
| *Chalcides bedriagai* | - | 0.1 | 0.1 | 0.1 | -8.1 | -10.6 | 1.0 |
| *Chalcides chalcides chalcides* | - | 0.2 | 0.2 | 0.2 | -33.5 | -43.7 | 1.0 |
| *Chalcides chalcides vittatus* | 3.9 | 11.7 | 1.5 | 6.4 | -218.4 | -1934.0 | 0.8 |
| *Chalcides ocellatus ocellatus* | 15.4 | 20.0 | 1.0 | 5.5 | -312.5 | -1460.0 | 0.5 |
| *Chalcides ocellatus tiligugu* | 20.8 | 37.7 | 0.5 | 6.4 | -133.8 | -1934.0 | 0.8 |
| *Chalcides parallelus* | - | 0.1 | 0.1 | 0.1 | -7.5 | -12.5 | 1.0 |
| *Chamaeleo chamaeleon* | 25.0 | 10.0 | 1.7 | 7.3 | -220.5 | -772.6 | 0.5 |
| *Coronella austriaca* | 23.1 | 1.1 | 0.2 | 0.5 | -38.8 | -124.6 | 1.0 |
| *Coronella girondica* | 3.3 | 0.2 | 0.0 | 0.1 | -19.2 | -53.9 | 1.0 |
| *Dalmatolacerta oxycephala* | 5.5 | 6.5 | 0.4 | 3.3 | -74.0 | -143.7 | 1.0 |
| *Dolichophis caspius* | 22.9 | 15.2 | 0.4 | 1.6 | -136.0 | -995.5 | 0.7 |
| *Dolichophis jugularis* | 10.2 | 3.5 | 0.4 | 1.1 | -254.2 | -772.6 | 0.5 |
| *Eirenis levantinus* | - | 1.3 | 1.3 | 1.3 | -313.9 | -774.8 | 0.5 |
| *Eirenis modestus* | 7.4 | 5.2 | 0.4 | 1.9 | -170.4 | -995.5 | 0.7 |
| *Elaphe quatuorlineata* | 22.4 | 17.0 | 0.5 | 2.7 | -111.0 | -396.8 | 0.8 |
| *Elaphe sauromates* | - | 0.1 | 0.1 | 0.1 | -21.3 | -26.2 | 1.0 |
| *Eryx jaculus* | 27.1 | 13.6 | 0.5 | 2.7 | -119.2 | -471.9 | 0.8 |
| *Euleptes europaea* | 20.5 | 24.0 | 0.2 | 7.4 | -155.8 | -1936.9 | 0.8 |
| *Eumeces schneiderii* | - | 1.1 | 1.1 | 1.1 | -477.9 | -772.6 | 0.1 |
| *Hemidactylus turcicus* | 103.9 | 111.5 | 0.5 | 7.3 | -211.8 | -1729.0 | 0.7 |
| *Hemorrhois algirus* | 4.6 | 6.7 | 1.7 | 6.0 | -183.8 | -544.9 | 0.6 |
| *Hemorrhois hippocrepis* | 18.3 | 8.3 | 1.2 | 3.1 | -437.1 | -1934.0 | 0.4 |
| *Hemorrhois nummifer* | 12.5 | 5.7 | 0.4 | 1.4 | -163.9 | -772.6 | 0.7 |
| *Heremites auratus* | 3.3 | 0.6 | 0.1 | 0.3 | -188.1 | -338.4 | 0.4 |
| *Heremites vittatus* | 21.7 | 1.3 | 0.3 | 1.1 | -404.7 | -772.6 | 0.3 |
| *Hierophis gemonensis* | 23.0 | 26.0 | 0.6 | 6.1 | -259.7 | -1480.1 | 0.6 |
| *Hierophis viridiflavus* | 42.1 | 55.1 | 0.6 | 14.4 | -193.4 | -2309.8 | 0.9 |
| *Lacerta bilineata* | 11.4 | 0.3 | 0.1 | 0.2 | -34.4 | -124.6 | 1.0 |
| *Lacerta trilineata* | 20.3 | 11.3 | 0.3 | 2.7 | -131.9 | -661.5 | 0.6 |
| *Lacerta viridis* | 13.7 | 1.2 | 0.2 | 0.5 | -41.1 | -58.5 | 1.0 |
| *Macroprotodon mauritanicus* | 10.8 | 8.2 | 2.1 | 4.6 | -1273.8 | -2804.9 | 0.5 |
| *Macrovipera lebetinus* | - | 1.3 | 1.3 | 1.3 | -313.9 | -774.8 | 0.5 |
| *Macrovipera schweizeri* | 0.8 | 0.4 | 0.1 | 0.3 | -108.8 | -230.6 | 0.7 |
| *Malpolon insignitus* | 45.8 | 14.9 | 0.6 | 2.3 | -96.8 | -772.6 | 0.8 |
| *Malpolon monspessulanus* | 11.2 | 0.6 | 0.1 | 0.1 | -25.7 | -65.5 | 1.0 |
| *Mediodactylus bartoni* | 2.0 | 0.9 | 0.1 | 0.4 | -175.2 | -462.7 | 0.5 |
| *Mediodactylus danilewskii* | 6.0 | 8.2 | 4.1 | 8.2 | -336.6 | -1480.1 | 0.5 |
| *Mediodactylus kotschyi* | 21.8 | 35.4 | 0.3 | 4.6 | -139.3 | -598.5 | 0.7 |
| *Mediodactylus oertzeni* | 2.7 | 3.4 | 0.7 | 1.5 | -654.5 | -1460.9 | 0.2 |
| *Mediodactylus orientalis* | 6.8 | 3.2 | 0.6 | 1.1 | -220.4 | -772.6 | 0.7 |
| *Mesalina olivieri* | 2.4 | 1.4 | 0.2 | 0.5 | -1.9 | -6.1 | 1.0 |
| *Montivipera xanthina* | 5.9 | 4.0 | 0.3 | 0.6 | -89.6 | -420.2 | 0.9 |
| *Natrix helvetica* | 9.7 | 3.8 | 1.0 | 3.2 | -105.6 | -421.0 | 0.8 |
| *Natrix maura* | 13.2 | 16.1 | 1.8 | 8.9 | -154.1 | -828.8 | 0.8 |
| *Natrix natrix* | 35.0 | 18.0 | 0.5 | 2.4 | -160.3 | -995.5 | 0.7 |
| *Natrix tessellata* | 17.9 | 4.1 | 0.7 | 1.7 | -146.3 | -661.5 | 0.6 |
| *Ophiomorus punctatissimus* | 0.1 | 0.3 | 0.2 | 0.3 | -76.3 | -217.4 | 0.9 |
| *Ophisops elegans* | 14.8 | 13.1 | 0.4 | 1.3 | -164.5 | -995.5 | 0.7 |
| *Phoenicolacerta laevis* | - | 0.1 | 0.1 | 0.1 | -7.4 | -11.9 | 1.0 |
| *Platyceps najadum* | 23.5 | 7.8 | 0.3 | 1.1 | -138.7 | -772.6 | 0.8 |
| *Podarcis cretensis* | 4.1 | 1.3 | 0.1 | 0.4 | -162.1 | -462.7 | 0.6 |
| *Podarcis erhardii* | 13.8 | 25.6 | 0.3 | 4.7 | -218.8 | -938.3 | 0.5 |
| *Podarcis filfolensis* | 2.6 | 6.3 | 1.6 | 4.3 | -253.8 | -543.1 | 0.3 |
| *Podarcis gaigae* | 0.9 | 0.9 | 0.1 | 0.5 | -108.8 | -226.6 | 0.7 |
| *Podarcis ionicus* | 4.0 | 2.4 | 0.3 | 0.8 | -327.3 | -1288.6 | 0.6 |
| *Podarcis latastei* | 0.3 | 0.2 | 0.1 | 0.1 | -38.9 | -69.7 | 1.0 |
| *Podarcis levendis* | 0.0 | 0.0 | 0.0 | 0.0 | -42.6 | -55.5 | 1.0 |
| *Podarcis lilfordi* | 3.3 | 0.7 | 0.0 | 0.2 | -29.7 | -82.8 | 1.0 |
| *Podarcis liolepis* | 4.9 | 0.8 | 0.3 | 0.7 | -57.4 | -80.8 | 1.0 |
| *Podarcis melisellensis* | 6.7 | 8.9 | 0.3 | 2.3 | -58.2 | -93.5 | 1.0 |
| *Podarcis milensis* | 1.5 | 1.8 | 0.3 | 1.0 | -343.7 | -711.1 | 0.3 |
| *Podarcis muralis* | 25.7 | 4.7 | 0.1 | 0.8 | -61.5 | -138.9 | 1.0 |
| *Podarcis peloponnesiacus* | 0.9 | 0.1 | 0.0 | 0.1 | -54.2 | -145.6 | 1.0 |
| *Podarcis pityusensis* | 2.4 | 1.9 | 0.1 | 1.4 | -200.9 | -706.9 | 0.7 |
| *Podarcis raffonei* | 1.0 | 1.5 | 0.5 | 0.7 | -910.2 | -1360.8 | 0.1 |
| *Podarcis siculus* | 10.7 | 56.3 | 0.5 | 18.6 | -163.2 | -1599.5 | 0.9 |
| *Podarcis tauricus* | 0.1 | 0.1 | 0.0 | 0.1 | -18.5 | -26.2 | 1.0 |
| *Podarcis tiliguerta* | 9.8 | 2.1 | 0.0 | 0.2 | -17.1 | -80.3 | 1.0 |
| *Podarcis vaucheri* | 1.5 | 0.1 | 0.0 | 0.1 | -7.6 | -12.5 | 1.0 |
| *Podarcis waglerianus* | 0.5 | 0.7 | 0.1 | 0.5 | -52.0 | -165.8 | 1.0 |
| *Psammodromus algirus* | 6.2 | 6.2 | 1.5 | 2.7 | -271.5 | -828.8 | 0.7 |
| *Pseudopus apodus* | 22.8 | 12.4 | 0.4 | 2.3 | -69.7 | -279.6 | 0.9 |
| *Ptyodactylus puiseuxi* | - | 0.1 | 0.1 | 0.1 | -7.4 | -11.9 | 1.0 |
| *Rhinechis scalaris* | 7.5 | 8.1 | 0.9 | 5.3 | -278.3 | -828.8 | 0.6 |
| *Saurodactylus mauritanicus* | 2.1 | 1.6 | 0.3 | 1.5 | -383.4 | -736.5 | 0.3 |
| *Scelarcis perspicillata* | 6.5 | 6.5 | 3.3 | 6.4 | -2332.5 | -2894.9 | 0.1 |
| *Stellagama stellio* | 19.6 | 26.1 | 0.8 | 9.8 | -224.5 | -1460.0 | 0.7 |
| *Stenodactylus mauritanicus* | 2.0 | 1.4 | 0.2 | 0.5 | -2.1 | -6.1 | 1.0 |
| *Tarentola mauritanica* | 68.7 | 98.8 | 0.8 | 18.1 | -208.6 | -1729.0 | 0.7 |
| *Telescopus fallax* | 46.4 | 51.8 | 0.8 | 14.1 | -179.9 | -1480.1 | 0.7 |
| *Timon lepidus* | 0.9 | 0.1 | 0.0 | 0.0 | -15.2 | -24.5 | 1.0 |
| *Timon pater* | - | 0.8 | 0.8 | 0.8 | -106.3 | -161.5 | 0.9 |
| *Trogonophis wiegmanni* | 0.0 | 0.1 | 0.0 | 0.1 | -8.1 | -12.5 | 1.0 |
| *Vipera ammodytes* | 22.1 | 10.1 | 0.4 | 2.6 | -118.0 | -286.9 | 0.8 |
| *Vipera aspis* | 7.3 | 1.5 | 0.5 | 1.2 | -107.8 | -199.5 | 0.9 |
| *Vipera latastei* | - | 0.7 | 0.7 | 0.7 | -59.1 | -80.8 | 1.0 |
| *Xerotyphlops vermicularis* | 23.9 | 9.6 | 0.4 | 2.4 | -155.7 | -772.6 | 0.7 |
| *Zamenis lineatus* | - | 0.1 | 0.1 | 0.1 | -112.1 | -127.3 | 1.0 |
| *Zamenis longissimus* | 13.4 | 1.2 | 0.1 | 0.3 | -51.8 | -112.1 | 1.0 |
| *Zamenis situlus* | 38.2 | 27.3 | 0.7 | 8.1 | -98.0 | -661.5 | 0.8 |

Appendix S2. Model M2

| Species | Modeled distance M2 | Average distance M2 | Max distance M2 | Average depth M2 | Max depth M2 | % above -150m M2 |
| --- | --- | --- | --- | --- | --- | --- |
| *Ablepharus budaki* | 1.5 | 0.7 | 1.4 | -293.4 | -774.6 | 0.6 |
| *Ablepharus kitaibelii* | 26.4 | 0.4 | 4.0 | -92.9 | -1017.6 | 0.9 |
| *Acanthodactylus boskianus* | 0.3 | 0.1 | 0.1 | -1.7 | -6.1 | 1.0 |
| *Acanthodactylus maculatus* | 0.4 | 0.4 | 0.4 | -2.7 | -4.0 | 1.0 |
| *Algyroides fitzingeri* | 0.6 | 0.1 | 0.2 | -6.7 | -36.3 | 1.0 |
| *Algyroides moreoticus* | 2.7 | 0.5 | 1.1 | -269.4 | -1351.0 | 0.7 |
| *Algyroides nigropunctatus* | 2.9 | 0.2 | 0.7 | -55.2 | -254.5 | 1.0 |
| *Anatololacerta anatolica* | 0.9 | 0.5 | 0.9 | -77.6 | -128.6 | 1.0 |
| *Anatololacerta finikensis* | 0.0 | 0.0 | 0.0 | -119.9 | -135.9 | 1.0 |
| *Anatololacerta pelasgiana* | 1.7 | 0.3 | 0.8 | -95.4 | -345.3 | 0.8 |
| *Anguis cephallonica* | 1.4 | 0.5 | 0.9 | -105.7 | -308.1 | 0.8 |
| *Anguis graeca* | 0.0 | 0.0 | 0.0 | -24.1 | -54.8 | 1.0 |
| *Anguis veronensis* | 0.0 | 0.0 | 0.0 | -4.0 | -5.1 | 1.0 |
| *Archaeolacerta bedriagae* | 0.5 | 0.1 | 0.2 | -5.8 | -36.3 | 1.0 |
| *Blanus strauchi* | 2.1 | 0.3 | 0.7 | -116.1 | -345.3 | 0.8 |
| *Chalcides bedriagai* | 0.1 | 0.1 | 0.1 | -8.1 | -10.6 | 1.0 |
| *Chalcides chalcides chalcides* | 0.2 | 0.2 | 0.2 | -33.5 | -43.7 | 1.0 |
| *Chalcides chalcides vittatus* | 12.4 | 1.5 | 7.2 | -158.2 | -2054.4 | 0.9 |
| *Chalcides ocellatus ocellatus* | 19.0 | 0.9 | 6.1 | -210.0 | -1731.4 | 0.8 |
| *Chalcides ocellatus tiligugu* | 35.3 | 0.5 | 7.2 | -92.4 | -2054.4 | 0.9 |
| *Chalcides parallelus* | 0.1 | 0.1 | 0.1 | -7.5 | -12.5 | 1.0 |
| *Chamaeleo chamaeleon* | 10.1 | 1.7 | 6.5 | -119.7 | -774.6 | 0.9 |
| *Coronella austriaca* | 1.1 | 0.2 | 0.5 | -38.8 | -124.6 | 1.0 |
| *Coronella girondica* | 0.2 | 0.0 | 0.1 | -19.2 | -53.9 | 1.0 |
| *Dalmatolacerta oxycephala* | 6.6 | 0.4 | 3.3 | -73.9 | -143.7 | 1.0 |
| *Dolichophis caspius* | 14.0 | 0.4 | 2.3 | -107.6 | -1017.6 | 0.9 |
| *Dolichophis jugularis* | 3.6 | 0.4 | 1.5 | -182.7 | -774.6 | 0.7 |
| *Eirenis levantinus* | 1.3 | 1.3 | 1.3 | -313.9 | -774.8 | 0.5 |
| *Eirenis modestus* | 5.1 | 0.4 | 2.0 | -139.8 | -1017.6 | 0.8 |
| *Elaphe quatuorlineata* | 15.4 | 0.4 | 3.0 | -79.0 | -539.9 | 1.0 |
| *Elaphe sauromates* | 0.1 | 0.1 | 0.1 | -21.3 | -26.2 | 1.0 |
| *Eryx jaculus* | 12.9 | 0.4 | 3.0 | -91.3 | -413.3 | 0.9 |
| *Euleptes europaea* | 23.5 | 0.2 | 8.1 | -106.1 | -2054.4 | 0.9 |
| *Eumeces schneiderii* | 1.5 | 1.5 | 1.5 | -294.5 | -774.6 | 0.6 |
| *Hemidactylus turcicus* | 95.5 | 0.5 | 7.5 | -121.4 | -2230.1 | 0.9 |
| *Hemorrhois algirus* | 7.8 | 2.0 | 7.1 | -74.6 | -413.3 | 0.9 |
| *Hemorrhois hippocrepis* | 21.9 | 3.1 | 17.8 | -102.4 | -828.8 | 0.9 |
| *Hemorrhois nummifer* | 6.0 | 0.4 | 1.5 | -130.3 | -774.6 | 0.8 |
| *Heremites auratus* | 0.5 | 0.1 | 0.3 | -131.4 | -345.3 | 0.7 |
| *Heremites vittatus* | 1.7 | 0.4 | 1.5 | -257.8 | -774.6 | 0.6 |
| *Hierophis gemonensis* | 18.5 | 0.5 | 5.7 | -95.6 | -1480.1 | 0.9 |
| *Hierophis viridiflavus* | 52.9 | 0.6 | 14.5 | -122.2 | -2228.9 | 0.9 |
| *Lacerta bilineata* | 0.3 | 0.1 | 0.2 | -34.4 | -124.6 | 1.0 |
| *Lacerta trilineata* | 11.6 | 0.4 | 2.8 | -94.4 | -661.5 | 0.9 |
| *Lacerta viridis* | 1.2 | 0.2 | 0.5 | -41.1 | -58.5 | 1.0 |
| *Macroprotodon mauritanicus* | 7.2 | 1.8 | 3.9 | -895.9 | -2818.8 | 0.7 |
| *Macrovipera lebetinus* | 1.3 | 1.3 | 1.3 | -313.9 | -774.8 | 0.5 |
| *Macrovipera schweizeri* | 0.3 | 0.1 | 0.2 | -92.6 | -258.0 | 0.8 |
| *Malpolon insignitus* | 14.3 | 0.6 | 2.3 | -78.7 | -774.6 | 0.9 |
| *Malpolon monspessulanus* | 0.6 | 0.1 | 0.1 | -25.7 | -65.5 | 1.0 |
| *Mediodactylus bartoni* | 0.9 | 0.1 | 0.4 | -185.6 | -462.7 | 0.5 |
| *Mediodactylus danilewskii* | 7.4 | 3.7 | 7.3 | -229.0 | -1248.0 | 0.7 |
| *Mediodactylus kotschyi* | 31.6 | 0.3 | 4.6 | -107.8 | -724.4 | 0.9 |
| *Mediodactylus oertzeni* | 3.2 | 0.6 | 1.3 | -515.5 | -1515.5 | 0.4 |
| *Mediodactylus orientalis* | 3.5 | 0.7 | 1.4 | -164.8 | -774.6 | 0.8 |
| *Mesalina olivieri* | 1.4 | 0.2 | 0.5 | -2.0 | -6.1 | 1.0 |
| *Montivipera xanthina* | 3.9 | 0.3 | 0.6 | -80.2 | -514.4 | 0.9 |
| *Natrix helvetica* | 3.8 | 1.0 | 3.4 | -76.6 | -430.4 | 1.0 |
| *Natrix maura* | 16.2 | 1.8 | 9.0 | -112.2 | -828.8 | 0.9 |
| *Natrix natrix* | 16.9 | 0.4 | 2.7 | -123.8 | -1017.6 | 0.9 |
| *Natrix tessellata* | 4.2 | 0.7 | 1.7 | -111.2 | -661.5 | 0.9 |
| *Ophiomorus punctatissimus* | 0.3 | 0.2 | 0.3 | -76.3 | -217.4 | 0.9 |
| *Ophisops elegans* | 13.2 | 0.4 | 1.5 | -135.4 | -1017.6 | 0.8 |
| *Phoenicolacerta laevis* | 0.1 | 0.1 | 0.1 | -7.4 | -11.9 | 1.0 |
| *Platyceps najadum* | 8.1 | 0.3 | 1.4 | -115.0 | -774.6 | 0.9 |
| *Podarcis cretensis* | 1.1 | 0.1 | 0.4 | -169.6 | -462.7 | 0.6 |
| *Podarcis erhardii* | 30.4 | 0.4 | 8.4 | -134.7 | -1203.3 | 0.8 |
| *Podarcis filfolensis* | 10.5 | 2.6 | 9.5 | -88.7 | -450.2 | 0.9 |
| *Podarcis gaigae* | 0.8 | 0.1 | 0.5 | -100.2 | -223.7 | 0.8 |
| *Podarcis ionicus* | 2.1 | 0.3 | 0.7 | -331.3 | -1351.0 | 0.7 |
| *Podarcis latastei* | 0.2 | 0.1 | 0.1 | -38.9 | -69.7 | 1.0 |
| *Podarcis levendis* | 0.0 | 0.0 | 0.0 | -42.6 | -55.5 | 1.0 |
| *Podarcis lilfordi* | 0.7 | 0.0 | 0.2 | -29.8 | -82.8 | 1.0 |
| *Podarcis liolepis* | 0.8 | 0.3 | 0.7 | -57.3 | -80.8 | 1.0 |
| *Podarcis melisellensis* | 9.0 | 0.3 | 2.3 | -58.2 | -93.5 | 1.0 |
| *Podarcis milensis* | 1.8 | 0.3 | 1.0 | -351.6 | -715.5 | 0.3 |
| *Podarcis muralis* | 4.6 | 0.1 | 0.8 | -61.4 | -138.9 | 1.0 |
| *Podarcis peloponnesiacus* | 0.1 | 0.0 | 0.1 | -54.2 | -145.6 | 1.0 |
| *Podarcis pityusensis* | 2.0 | 0.1 | 1.4 | -169.1 | -706.9 | 0.8 |
| *Podarcis raffonei* | 1.1 | 0.4 | 0.5 | -757.3 | -1386.1 | 0.1 |
| *Podarcis siculus* | 54.7 | 0.5 | 18.6 | -134.7 | -2226.5 | 0.9 |
| *Podarcis tauricus* | 0.1 | 0.0 | 0.1 | -18.5 | -26.2 | 1.0 |
| *Podarcis tiliguerta* | 2.1 | 0.0 | 0.2 | -17.0 | -80.3 | 1.0 |
| *Podarcis vaucheri* | 0.1 | 0.0 | 0.1 | -7.6 | -12.5 | 1.0 |
| *Podarcis waglerianus* | 0.8 | 0.1 | 0.5 | -51.3 | -165.1 | 1.0 |
| *Psammodromus algirus* | 6.1 | 1.5 | 2.7 | -180.0 | -828.8 | 0.8 |
| *Pseudopus apodus* | 11.7 | 0.4 | 2.3 | -60.9 | -258.5 | 1.0 |
| *Ptyodactylus puiseuxi* | 0.1 | 0.1 | 0.1 | -7.4 | -11.9 | 1.0 |
| *Rhinechis scalaris* | 6.8 | 0.8 | 5.3 | -167.5 | -828.8 | 0.8 |
| *Saurodactylus mauritanicus* | 0.7 | 0.1 | 0.6 | -520.6 | -1069.1 | 0.4 |
| *Scelarcis perspicillata* | 18.4 | 9.2 | 18.3 | -108.5 | -828.8 | 0.9 |
| *Stellagama stellio* | 28.9 | 0.9 | 13.6 | -135.0 | -1460.0 | 0.8 |
| *Stenodactylus mauritanicus* | 1.4 | 0.2 | 0.5 | -2.1 | -6.1 | 1.0 |
| *Tarentola mauritanica* | 91.5 | 0.7 | 23.0 | -107.9 | -2230.1 | 0.9 |
| *Telescopus fallax* | 49.8 | 0.8 | 17.1 | -99.3 | -1480.1 | 0.9 |
| *Timon lepidus* | 0.1 | 0.0 | 0.0 | -15.2 | -24.5 | 1.0 |
| *Timon pater* | 0.8 | 0.8 | 0.8 | -106.3 | -161.5 | 0.9 |
| *Trogonophis wiegmanni* | 0.1 | 0.0 | 0.1 | -8.1 | -12.5 | 1.0 |
| *Vipera ammodytes* | 8.7 | 0.3 | 2.9 | -84.6 | -258.5 | 1.0 |
| *Vipera aspis* | 1.7 | 0.6 | 1.4 | -106.2 | -211.9 | 0.9 |
| *Vipera latastei* | 0.7 | 0.7 | 0.7 | -59.1 | -80.8 | 1.0 |
| *Xerotyphlops vermicularis* | 9.5 | 0.4 | 2.4 | -126.0 | -774.6 | 0.9 |
| *Zamenis lineatus* | 0.1 | 0.1 | 0.1 | -112.1 | -127.3 | 1.0 |
| *Zamenis longissimus* | 1.2 | 0.1 | 0.3 | -51.8 | -112.1 | 1.0 |
| *Zamenis situlus* | 27.1 | 0.6 | 8.5 | -72.6 | -661.5 | 0.9 |

Appendix S2. M3.

| Species | Modeled distance M3 | Average distance M3 | Max distance M3 | Average depth M3 | Max depth M3 | % above -150m M3 |
| --- | --- | --- | --- | --- | --- | --- |
| *Ablepharus budaki* | 1.3 | 0.6 | 1.2 | -221.2 | -871.2 | 0.6 |
| *Ablepharus kitaibelii* | 23.5 | 0.3 | 4.0 | -68.9 | -1292.0 | 0.9 |
| *Acanthodactylus boskianus* | 0.2 | 0.1 | 0.1 | -0.7 | -5.5 | 1.0 |
| *Acanthodactylus maculatus* | 0.4 | 0.4 | 0.4 | -1.8 | -6.8 | 1.0 |
| *Algyroides fitzingeri* | 0.6 | 0.1 | 0.2 | -6.9 | -36.3 | 1.0 |
| *Algyroides moreoticus* | 3.5 | 0.7 | 2.3 | -169.4 | -1927.4 | 0.9 |
| *Algyroides nigropunctatus* | 2.9 | 0.2 | 0.7 | -25.9 | -254.9 | 1.0 |
| *Anatololacerta anatolica* | 0.9 | 0.4 | 0.9 | -37.3 | -124.0 | 1.0 |
| *Anatololacerta finikensis* | 0.0 | 0.0 | 0.0 | -119.9 | -135.9 | 1.0 |
| *Anatololacerta pelasgiana* | 2.3 | 0.4 | 0.7 | -60.5 | -418.5 | 0.8 |
| *Anguis cephallonica* | 1.0 | 0.3 | 0.7 | -68.7 | -258.3 | 0.9 |
| *Anguis graeca* | 0.0 | 0.0 | 0.0 | -24.1 | -54.8 | 1.0 |
| *Anguis veronensis* | 0.0 | 0.0 | 0.0 | -4.0 | -5.1 | 1.0 |
| *Archaeolacerta bedriagae* | 0.5 | 0.1 | 0.2 | -5.6 | -36.3 | 1.0 |
| *Blanus strauchi* | 2.4 | 0.3 | 0.7 | -67.4 | -418.5 | 0.9 |
| *Chalcides bedriagai* | 0.1 | 0.1 | 0.1 | -8.3 | -11.0 | 1.0 |
| *Chalcides chalcides chalcides* | 0.1 | 0.1 | 0.1 | -35.1 | -44.6 | 1.0 |
| *Chalcides chalcides vittatus* | 25.0 | 3.1 | 23.0 | -2.5 | -478.8 | 1.0 |
| *Chalcides ocellatus ocellatus* | 33.0 | 1.7 | 21.3 | -18.1 | -1292.0 | 0.9 |
| *Chalcides ocellatus tiligugu* | 29.2 | 0.4 | 5.9 | -66.4 | -2194.1 | 0.9 |
| *Chalcides parallelus* | 0.0 | 0.0 | 0.0 | -8.7 | -12.5 | 1.0 |
| *Chamaeleo chamaeleon* | 7.2 | 1.2 | 5.4 | -94.3 | -871.2 | 0.9 |
| *Coronella austriaca* | 0.9 | 0.1 | 0.4 | -36.6 | -124.6 | 1.0 |
| *Coronella girondica* | 0.2 | 0.0 | 0.1 | -19.2 | -53.9 | 1.0 |
| *Dalmatolacerta oxycephala* | 4.6 | 0.3 | 2.4 | -24.8 | -140.2 | 1.0 |
| *Dolichophis caspius* | 14.0 | 0.4 | 2.6 | -71.8 | -1292.0 | 0.9 |
| *Dolichophis jugularis* | 3.1 | 0.3 | 1.3 | -158.0 | -871.2 | 0.7 |
| *Eirenis levantinus* | 1.3 | 1.3 | 1.3 | -221.3 | -871.2 | 0.6 |
| *Eirenis modestus* | 4.4 | 0.3 | 2.2 | -131.2 | -1292.0 | 0.8 |
| *Elaphe quatuorlineata* | 12.0 | 0.3 | 3.2 | -47.0 | -596.4 | 1.0 |
| *Elaphe sauromates* | 0.1 | 0.1 | 0.1 | -21.3 | -26.2 | 1.0 |
| *Eryx jaculus* | 10.9 | 0.4 | 2.9 | -59.1 | -478.8 | 0.9 |
| *Euleptes europaea* | 21.3 | 0.2 | 8.9 | -65.6 | -2194.1 | 0.9 |
| *Eumeces schneiderii* | 1.3 | 1.3 | 1.3 | -221.3 | -871.2 | 0.6 |
| *Hemidactylus turcicus* | 81.1 | 0.4 | 7.2 | -106.5 | -2230.1 | 0.9 |
| *Hemorrhois algirus* | 6.8 | 1.7 | 6.2 | -51.9 | -478.8 | 1.0 |
| *Hemorrhois hippocrepis* | 24.3 | 3.5 | 19.5 | -75.0 | -1675.7 | 0.9 |
| *Hemorrhois nummifer* | 6.6 | 0.5 | 2.0 | -74.3 | -871.2 | 0.9 |
| *Heremites auratus* | 0.5 | 0.1 | 0.3 | -122.1 | -368.2 | 0.7 |
| *Heremites vittatus* | 1.4 | 0.4 | 1.3 | -192.6 | -871.2 | 0.6 |
| *Hierophis gemonensis* | 29.5 | 0.7 | 15.9 | -36.6 | -1354.2 | 0.9 |
| *Hierophis viridiflavus* | 49.4 | 0.6 | 18.4 | -62.9 | -2230.1 | 0.9 |
| *Lacerta bilineata* | 0.3 | 0.1 | 0.1 | -35.3 | -124.6 | 1.0 |
| *Lacerta trilineata* | 11.0 | 0.3 | 3.1 | -59.1 | -877.7 | 0.9 |
| *Lacerta viridis* | 1.0 | 0.2 | 0.5 | -39.3 | -64.1 | 1.0 |
| *Macroprotodon mauritanicus* | 26.4 | 6.6 | 23.0 | -1.1 | -828.8 | 0.9 |
| *Macrovipera lebetinus* | 1.3 | 1.3 | 1.3 | -221.3 | -871.2 | 0.6 |
| *Macrovipera schweizeri* | 0.3 | 0.1 | 0.3 | -72.8 | -268.6 | 0.9 |
| *Malpolon insignitus* | 12.1 | 0.5 | 1.8 | -56.7 | -871.2 | 0.9 |
| *Malpolon monspessulanus* | 0.5 | 0.0 | 0.2 | -17.2 | -51.5 | 1.0 |
| *Mediodactylus bartoni* | 0.7 | 0.1 | 0.3 | -190.1 | -575.7 | 0.5 |
| *Mediodactylus danilewskii* | 21.8 | 10.9 | 21.8 | 16.0 | -1354.2 | 1.0 |
| *Mediodactylus kotschyi* | 33.8 | 0.3 | 10.0 | -54.8 | -740.3 | 0.9 |
| *Mediodactylus oertzeni* | 3.2 | 0.6 | 1.5 | -488.7 | -2194.3 | 0.5 |
| *Mediodactylus orientalis* | 2.5 | 0.5 | 1.2 | -137.0 | -871.2 | 0.8 |
| *Mesalina olivieri* | 1.2 | 0.1 | 0.5 | -1.5 | -5.8 | 1.0 |
| *Montivipera xanthina* | 3.6 | 0.3 | 0.6 | -72.8 | -545.8 | 0.9 |
| *Natrix helvetica* | 3.6 | 0.9 | 3.4 | -42.1 | -460.8 | 1.0 |
| *Natrix maura* | 15.4 | 1.7 | 9.9 | -93.6 | -1675.7 | 0.9 |
| *Natrix natrix* | 14.3 | 0.4 | 2.9 | -94.9 | -1292.0 | 0.9 |
| *Natrix tessellata* | 4.3 | 0.7 | 1.6 | -85.5 | -877.7 | 0.9 |
| *Ophiomorus punctatissimus* | 0.2 | 0.1 | 0.2 | -99.5 | -313.6 | 0.7 |
| *Ophisops elegans* | 12.2 | 0.4 | 1.7 | -113.3 | -1292.0 | 0.8 |
| *Phoenicolacerta laevis* | 0.0 | 0.0 | 0.0 | -7.3 | -11.9 | 1.0 |
| *Platyceps najadum* | 7.5 | 0.3 | 1.2 | -83.5 | -871.2 | 0.9 |
| *Podarcis cretensis* | 0.9 | 0.1 | 0.3 | -170.9 | -575.7 | 0.6 |
| *Podarcis erhardii* | 27.5 | 0.3 | 8.5 | -97.7 | -1350.5 | 0.9 |
| *Podarcis filfolensis* | 11.6 | 2.9 | 10.3 | -50.9 | -521.2 | 0.9 |
| *Podarcis gaigae* | 0.8 | 0.1 | 0.5 | -96.0 | -223.7 | 0.8 |
| *Podarcis ionicus* | 2.0 | 0.2 | 0.7 | -304.2 | -1927.4 | 0.8 |
| *Podarcis latastei* | 0.2 | 0.1 | 0.1 | -38.5 | -69.7 | 1.0 |
| *Podarcis levendis* | 0.1 | 0.1 | 0.1 | -44.0 | -55.5 | 1.0 |
| *Podarcis lilfordi* | 0.5 | 0.0 | 0.2 | -22.9 | -68.0 | 1.0 |
| *Podarcis liolepis* | 0.6 | 0.2 | 0.5 | -58.1 | -88.0 | 1.0 |
| *Podarcis melisellensis* | 5.4 | 0.2 | 0.9 | -32.8 | -90.9 | 1.0 |
| *Podarcis milensis* | 1.5 | 0.2 | 1.0 | -222.6 | -683.6 | 0.4 |
| *Podarcis muralis* | 3.1 | 0.1 | 0.6 | -29.6 | -137.5 | 1.0 |
| *Podarcis peloponnesiacus* | 0.0 | 0.0 | 0.0 | -36.5 | -131.8 | 1.0 |
| *Podarcis pityusensis* | 1.8 | 0.1 | 1.3 | -172.5 | -711.1 | 0.7 |
| *Podarcis raffonei* | 0.9 | 0.3 | 0.5 | -477.1 | -1361.9 | 0.4 |
| *Podarcis siculus* | 46.2 | 0.4 | 16.3 | -111.8 | -2226.5 | 0.9 |
| *Podarcis tauricus* | 0.1 | 0.0 | 0.1 | -18.5 | -26.2 | 1.0 |
| *Podarcis tiliguerta* | 1.8 | 0.0 | 0.2 | -14.8 | -84.6 | 1.0 |
| *Podarcis vaucheri* | 0.1 | 0.0 | 0.0 | -7.9 | -12.7 | 1.0 |
| *Podarcis waglerianus* | 0.7 | 0.1 | 0.4 | -44.8 | -165.1 | 1.0 |
| *Psammodromus algirus* | 5.9 | 1.5 | 3.4 | -228.1 | -1675.7 | 0.8 |
| *Pseudopus apodus* | 8.5 | 0.3 | 1.5 | -33.5 | -258.3 | 1.0 |
| *Ptyodactylus puiseuxi* | 0.0 | 0.0 | 0.0 | -7.3 | -11.9 | 1.0 |
| *Rhinechis scalaris* | 6.3 | 0.7 | 4.3 | -278.0 | -1675.7 | 0.7 |
| *Saurodactylus mauritanicus* | 0.6 | 0.1 | 0.5 | -559.3 | -1087.3 | 0.3 |
| *Scelarcis perspicillata* | 18.1 | 9.0 | 18.0 | -24.1 | -828.8 | 0.9 |
| *Stellagama stellio* | 38.5 | 1.1 | 18.7 | -20.1 | -1367.8 | 0.9 |
| *Stenodactylus mauritanicus* | 1.1 | 0.2 | 0.5 | -1.4 | -5.8 | 1.0 |
| *Tarentola mauritanica* | 80.5 | 0.7 | 24.0 | -56.8 | -2230.1 | 0.9 |
| *Telescopus fallax* | 46.3 | 0.7 | 18.5 | -57.4 | -1927.4 | 0.9 |
| *Timon lepidus* | 0.1 | 0.0 | 0.0 | -15.9 | -24.5 | 1.0 |
| *Timon pater* | 0.5 | 0.5 | 0.5 | -95.4 | -208.8 | 0.9 |
| *Trogonophis wiegmanni* | 0.1 | 0.0 | 0.0 | -7.9 | -12.7 | 1.0 |
| *Vipera ammodytes* | 7.3 | 0.3 | 2.5 | -43.4 | -310.9 | 1.0 |
| *Vipera aspis* | 1.0 | 0.3 | 1.0 | -75.2 | -215.7 | 0.9 |
| *Vipera latastei* | 0.5 | 0.5 | 0.5 | -61.0 | -88.0 | 1.0 |
| *Xerotyphlops vermicularis* | 7.4 | 0.3 | 2.2 | -87.3 | -871.2 | 0.9 |
| *Zamenis lineatus* | 0.1 | 0.1 | 0.1 | -112.1 | -127.3 | 1.0 |
| *Zamenis longissimus* | 0.9 | 0.1 | 0.2 | -38.6 | -86.2 | 1.0 |
| *Zamenis situlus* | 22.9 | 0.5 | 8.1 | -45.8 | -877.7 | 0.9 |
